# Factors Contributing to Anastomotic Leakage Following Colorectal Surgery: Why, When, and Who Leaks?

**DOI:** 10.7759/cureus.29964

**Published:** 2022-10-05

**Authors:** Shravani Sripathi, Mashal I Khan, Naomi Patel, Roja T Meda, Surya P Nuguru, Sriker Rachakonda

**Affiliations:** 1 General Surgery, Bhaskar Medical College, Hyderabad, IND; 2 Internal Medicine, Khyber Girls Medical College, Peshawar, PAK; 3 Research, Smt. Nathiba Hargovandas Lakhmichand Municipal Medical College, Ahmedabad, IND; 4 Miscellaneous, Narayana Medical College, Nellore, IND; 5 Internal Medicine, Kamineni Academy of Medical Sciences and Research Center, Hyderabad, IND; 6 Surgery, OO Bogomolets National Medical University, Kyiv, UKR

**Keywords:** colorectal cancer, colorectal surgery, anastomotic dehiscence, anastomotic leak, anastomosis, risk factors

## Abstract

Anastomotic leakage is a common yet one of the most feared complications following colorectal surgery. Dehiscence of the anastomosis can result in fatal complications such as peritonitis, abscess formation, and sepsis, thereby increasing morbidity and mortality, cost and length of hospital stay. Multiple factors contribute to the development of anastomotic dehiscence. Several studies have been published identifying various risk factors that may play a role in causing AL. Our study reviewed prospective and retrospective studies and summarized the risk factors into three categories: preoperative, intraoperative, and postoperative. Among these are various risk factors such as age, gender, comorbidities, American Society of Anesthesiologists (ASA) scores, operative time, smoking, alcohol use, obesity, nutritional status, mechanical bowel preparation, and steroid use. It is crucial for surgeons to have a thorough understanding of the risk factors associated with anastomotic leakage to identify patients at high risk preoperatively. It may also be relevant to intraoperative decision-making when establishing an anastomosis, such as considering proximal diversion or placing a drain if such high-risk features are present. Knowing high-risk features also helps to detect leaks as early as possible postoperatively.

## Introduction and background

Colorectal surgery is performed for various malignant and benign conditions such as colorectal cancer, inflammatory bowel disease, bowel obstruction, recurrent diverticulitis, trauma, and ischemia, often requiring bowel resection [1]. Following resection, the healthy sections of the intestine are reconnected by an anastomosis to ensure gastrointestinal continuity [2]. Bowel anastomosis has been practiced ever since the 19th century [2]. Over the last 200 years, the gastrointestinal anastomosis procedure has evolved from being a life-threatening surgery to a safe and commonly performed procedure [2].

One of the most significant surgical complications of gastrointestinal anastomosis is anastomotic leakage (AL), leading to an increase in the risk of morbidity and mortality [1]. The International Study Group of Rectal Cancer (ISREC) defined AL as "a communication between the intra- and extra-luminal compartments owing to a defect of the integrity of the intestinal wall at the anastomotic site" [3]. Anastomotic defects can lead to complications such as peritonitis, abscess formation, and sepsis, all of which can be fatal [4]. The incidence of anastomotic leak in colorectal surgery is up to 20% [5]. The mortality rates associated with AL range from 10% to 20% [6]. AL was found to have a detrimental influence on overall survival in a meta-analysis that included a total of 154,981 patients [7]. Furthermore, another systematic review and meta-analysis involving 78’434 colorectal cancer patients reported that AL was related to a higher likelihood of local recurrence [risk ratio (RR) 1.90] and a substantially lower overall survival (RR 1.36) [8]. The occurrence of AL following colorectal surgery is related to several factors (Table 1), such as preoperative, intra-operative, and postoperative factors [9].

**Table 1:** Factors associated with increased risk of anastomotic leakage (AL) after colorectal surgeries

Preoperative	Intraoperative	Postoperative
Age	Level of anastomosis	Intestinal microbes
Gender	Vascularization of intestinal segments	Postoperative diet
Obesity	Operative time	
Preoperative nutritional status	Intraoperative blood loss	
Smoking	Blood transfusion	
Alcohol	Diverting stoma	
American Society of Anesthesiologists (ASA) score	Prophylactic drains	
Steroid use		
Neoadjuvant chemoradiotherapy		
Mechanical bowel preparation		

Our review article aims to provide an overview of the current literature on factors playing a role in causing AL in patients undergoing colorectal surgeries. Risk factors, when assessed, assist physicians in identifying patients at high risk, which has a great deal of clinical significance and ultimately improves patient outcomes.

## Review

For this review article, electronic databases PubMed and Google Scholar were used. A literature search using the keywords colorectal surgery, anastomotic leak, dehiscence, and risk factors was carried out. All papers not in the English language were excluded. Prospective, retrospective, randomized studies, and meta-analyses were all included in this review. We categorized the factors that may potentially contribute to the leakage of anastomosis into three categories- preoperative, intraoperative, and postoperative factors.

Preoperative factors

Age

Very few studies have shown that age was significantly associated with AL [10-12]. A prospective study including patients undergoing rectal cancer surgery conducted by Lin et al. demonstrated age >70 years as an independent risk factor for AL with OR 2.17 (95% CI: 1.21-3.88) and p-value = 0.009 [12]. Also, a retrospective study published in 2008 by Jung et al., which included 1391 patients who underwent rectal cancer surgery, found that age >60 years was an independent risk factor for AL with a hazard ratio (HR) of 2.32 (95% CI: 1.12-7.83) [11]. But, in most of the studies reviewed, the patient's age did not have a statistically significant association with the AL rate [13-18]. In a meta-analysis conducted by Pommergaard et al. in Sweden, including 110,272 colorectal cancer patients, found a pooled OR of 0.99 (95% CI: 0.89-1.10), suggesting that age was not significantly associated with AL [19]. However, in a retrospective study conducted by Bakker et al. including 15667 colorectal cancer patients, it was found that increasing age was a significant risk factor for death after AL, with 30.1% deaths in patients aged>80 years compared to 18.3% deaths in age 65-80 years and 5.2% deaths in age < 65 years with a p-value of <0.001 [18].

Gender

Male gender has often been reported as an independent risk factor for leakage in colorectal [16,17,20-22]. A multivariate analysis performed by Lipska et al. in New Zealand revealed that among patients undergoing anastomoses involving the colon and rectum, male patients had a higher incidence of AL than female patients, with an OR of 3.5 [16]. Park et al., in their retrospective study of 1609 patients with rectal cancer, showed that the male sex was significantly associated with AL with an HR of 3.468 (95% CI: 1.646-7.305; p=0.001) [20]. A study by Hamabe et al. in Japan, including 296 patients undergoing Laparoscopic low anterior resection with anastomosis using the double stapling technique, demonstrated that men were at significantly higher risk of AL with OR=18.0 (95% CI: 2.4-138; p=0.0053) [21]. Anatomically, males have a narrower pelvis, leading to more difficult resections in both open and laparoscopic colorectal surgeries [22]. Low rectal procedures are likely to have this gender difference due to the more challenging dissection and anastomoses [17,20,21]. Also, androgen-related differences in intestinal microcirculation may contribute to anastomosis healing [23].

Obesity

Several studies have demonstrated that obesity measured by body mass index (BMI) is an independent indicator of an anastomotic leak [24-27]. In a study conducted by Biondo et al. including patients who underwent left colonic resection and primary anastomosis, it was found that obesity with BMI >30 is an independent risk factor associated with AL [27]. In another prospective study by Frasson et al. including 3193 colorectal cancer patients, it was demonstrated that patients with BMI >30 are at an increased risk of AL with an OR of 2.7 (95% CI: 1.4-5.1; p=0.003) [24]. In addition, a CT scan measurement of visceral fat provides a more sensitive indicator than the BMI of anastomotic dehiscence [28]. Yang et al. conducted a meta-analysis in which it was found that in patients undergoing colorectal surgery, visceral obesity is associated with complications including longer operative time, more conversion to open procedure, increased morbidity, more surgical site infections, and higher anastomotic leaks [29]. The association between obesity and AL has been explained by several hypotheses: obesity may indicate a problem with the tissue structure and healing, or a higher abdominal pressure may negatively affect anastomosis microcirculation. An additional assumption is that obese patients have a thicker mesocolon, making anastomosis more difficult [24].

Preoperative Nutrition

Malnutrition negatively impacts anastomotic healing by impairing collagen synthesis or fibroblast proliferation [30]. A retrospective study conducted by Kang et al. involving 72,055 patients who underwent elective anterior resection showed that preoperative weight loss and malnutrition (OR= 2.81; 95% CI: 2.32-3.40), fluid and electrolyte disorders (OR= 1.79; 95% CI: 1.58-2.03) were significantly associated with AL [31]. Zhu et al. conducted a study in which preoperative nutritious status was mainly evaluated by hemoglobin and albumin and found that anemia (hemoglobin < 100 g/L) or hypoproteinemia (albumin ≤ 32 g/L) was found to be significantly associated with AL [32]. Similarly, in a case-control study conducted by Telem et al. which included patients undergoing colorectal resection, it was demonstrated that preoperative albumin level <3.5 mg/dL increased the risk of AL with OR of 2.8 (95% CI: 1.3-5.1; P = 0.03) [14].

Smoking and Alcohol

Smoking and alcohol abuse are significant factors contributing to AL [17,33-35]. Researchers have found that smoking impairs tissue healing and increases AL [34]. Sørensen et al. conducted a retrospective study including patients who underwent colonic or rectal resection with anastomosis that showed an increased risk of AL in smokers compared to non-smokers with a RR of 3.18 and alcohol abusers compared to non-alcoholics with a RR of 7.18 [33]. Another prospective study in Germany demonstrated smoking and alcohol use as independent risk factors for AL [17]. An excessive amount of alcohol consumption may reflect poor nutritional status. In smokers, vascular ischemia from nicotine-induced vasoconstriction and microthromboses, in combination with carbon monoxide-induced cellular hypoxia, inhibits anastomotic circulation [35].

American Society of Anesthesiologists (ASA) Score

The American Society of Anesthesiologists (ASA) Classification is a tool used by anesthesiologists to evaluate the preoperative health condition of a patient before surgery to predict the operative risks [36]. The ASA score is classified on a scale ranging from I to VI [36]. A high ASA grade(≥3) has been shown to be an important risk factor for AL [15,18,37]. Bakker et al. conducted a study that demonstrated higher rates of AL in colorectal cancer patients with ASA scores III and IV (9.2%) compared with ASA scores I and II (7.1%) with a significant p-value of < 0.001 [18]. In a retrospective study by Choi et al. including 1417 colorectal cancer patients, it was found that a high ASA score (III to V) is a significant risk factor for leakage of the anastomosis with OR 5.6 (95%CI: 1.6-15.3; p=0.04) [37]. In a prospective study including all colorectal procedures conducted over 40 months by Buchs et al. in Switzerland, it was observed that the risk of leakage increased 2.5 times for each unit increase of ASA score [15].

Steroid Use

Chronic steroid use refers to the use of long-term, systemic steroids, which require the administration of additional stress-dose steroids during surgery [13]. In a randomized control trial study in a rat model conducted by Mantzoros et al., it was demonstrated that colonic anastomotic healing was affected in the steroid treatment group with statistically significant higher leakage rates in the treatment group than in the control group (p<0.001) [38]. It was postulated that steroid use causes a significant decrease in inflammatory cell infiltration and collagen deposition affecting anastomotic healing [38]. Slieker et al. conducted a prospective study including 259 patients undergoing left-sided colorectal anastomoses that showed that the incidence of AL was 50% in patients on long-term steroid use (p=0.002) and 19% in patients taking corticosteroids perioperatively (p=0.001) [39]. In a prospective study conducted over four years in Japan, it was found that long-term steroid use was a significant risk factor for AL in colorectal cancer surgery, with a leakage rate of 11.8% vs. 2.4% in patients with and without steroid use, respectively [13]. Chronic use of corticosteroids can increase the risk of AL, especially when combined with other immunosuppressive drugs [13].

Neoadjuvant Chemoradiotherapy

Neoadjuvant chemoradiotherapy is a part of multimodality treatment for patients with locally advanced primary and recurrent rectal cancers [40]. Preoperative chemotherapy is known to increase the risk of AL [41]. There are also many harmful effects of radiotherapy on intestinal tissue and wound healing, and it has long been known to contribute as a risk factor for AL [42]. However, various studies reviewed have found conflicting results. In a retrospective study conducted in Korea by Park et al., including 1609 patients with rectal cancer, it was found that preoperative chemoradiation was not associated with an increased risk of AL on univariate analysis. However, in a subgroup of patients without a protective stoma, it was demonstrated that preoperative chemoradiation increased the risk of AL with an HR of 6.284 (95%CI: 2.829-13.961; p<0.001) [20]. In contrast, a retrospective study conducted by Chang et al. using propensity score matching analysis showed that preoperative chemoradiotherapy did not increase the risk of AL after rectal cancer resection [43]. A meta-analysis conducted by Qu et al. indicated that preoperative chemotherapy was associated with the development of AL (OR 1.67, 95 % CI 1.10-2.55, P = 0.02) [41]. However, a randomized trial conducted by Marijnen et al. found that there was no significant difference in AL rates with or without preoperative radiotherapy [44]. Due to inconsistent results, there should certainly be more studies designed to determine the effect of preoperative chemoradiation in AL.

Mechanical Bowel Preparation

Mechanical bowel preparation aims to reduce bowel contents before colorectal surgery, which are sources of infectious bacteria that increase the risk of surgical site infections and AL [45]. According to a meta-analysis conducted by Rollins et al., it was concluded that the use of mechanical bowel preparation in elective colorectal surgery did not impact the incidence of postoperative complications when compared with those who did not undergo preparation (OR = 0.90, 95%CI: 0.74 to 1.10, P = 0.32) [46]. Scarborough et al. conducted a study including 4999 patients which showed that compared to no preoperative bowel preparation, combination of mechanical bowel preparation and oral antibiotics significantly reduced the postoperative complications such as surgical site infections (3.2% vs 9.0%, P < 0.001), AL (2.8% vs 5.7%, P = 0.001), and hospital readmission (5.5% vs 8.0%, P = 0.03) [45]. In a retrospective study performed by Garfinkle et al., that included 40,446 patients undergoing elective colectomy, it was reported that compared with oral antibiotics alone, a combined regimen of oral antibiotics and mechanical bowel preparation offered no superiority in postoperative outcomes [47]. Further studies are needed to give a clearer picture of the effects of mechanical bowel preparation on AL.

Intraoperative factors

Level of Anastomosis

Among the most important factors predicting leakage is the distance of the anastomosis from the anal verge [48]. An anastomosis of 5 cm or less from the anal verge is defined as a low rectal anastomosis [48]. In a case-control study by Jestin et al. including rectal cancer patients, it was shown that anastomosis level ≤ 6cm was an independent risk factor significantly associated with AL both on univariate (OR=1.38; 95%CI: 1.08-1.77) and multivariate analysis (OR=1.39; 95%CI: 1.01-1.90) [49]. In a retrospective study conducted by Choi et al. including patients undergoing rectal resection, it was demonstrated that the AL rate was 10 times higher (20.6% vs. 2.3%) when the anastomotic region was located within 5 cm of the anal verge [48]. Also, in a meta-analysis conducted by Pommergaard et al., including 110,272 colorectal cancer patients, it was found that a low rectal anastomosis was associated with a high risk of leakage with an OR of 3.26 (95% CI: 2.31-4.62) [19]. There is a hypothesis that low anastomosis is associated with technical difficulties that result in tissue trauma, tension, or poor blood supply [9]. The collateral arteries in the rectal region are few and highly variable; therefore, unrecognized damage to small arteries during resection may adversely affect perfusion, resulting in AL [50].

Vascularization of Intestinal Segments

It is vital that blood flow to the intestinal segments is adequate during the healing process of the sutures [51]. Therefore, surgeons usually assess intestinal perfusion during surgery according to their experience by assessing the color of the intestinal segment, pulsations, and the presence of bleeding. These observations are subjective and may lead to misinterpretations [52]. Doppler ultrasound was routinely used intraoperatively because of its low cost and simple technique and was considered more reliable than doing clinical assessment alone [53]. However, in a study conducted by Dyess et al., doppler ultrasonography had a high rate of false-positive and false-negative results. It was found to be an unreliable tool for assessing intestinal perfusion [54]. Recently, it was suggested that a real-time assessment of intestinal perfusion could be obtained utilizing near-infrared (NIR) fluorescence technology with indocyanine green (ICG) [55]. In a study conducted by Kudszus et al. in patients undergoing colorectal cancer resections, it was reported that intraoperative fluorescence imaging resulted in the reduction of AL by 4% in the study group compared with the control group [55]. Similarly, a multi-institutional prospective study conducted by Jafari et al. demonstrated that fluorescence imaging changed surgical plans in 8% of patients, and there were no anastomotic leaks in those patients [56].

Operative Time

Many factors play a role in influencing the duration of operation, such as obesity, type of surgery (open vs. laparoscopic), surgeon's experience, presence of adhesions due to prior abdominal surgery, and other intraoperative complications [57]. Prolonged operative time increases the risk of bacterial exposure and tissue damage causing inflammation and resulting in complications such as ischemia, sepsis, and AL [9]. In a retrospective analysis conducted by Midura et al., which included 13,684 patients who underwent segmental colectomy, it was reported that operative time > 3 hours was associated with increased AL (OR=1.50; 95% CI: 1.19-1.90; p= 0.001) [58]. In another prospective study by Konishi et al., it was found that ≥ 4 hours of surgery duration was associated with AL with OR 9.9 (95% CI: 1.7-186.2; p=0.034) [13]. Studies have shown that prolonged operative time significantly increases the risk of developing AL, with the threshold varying from 180 to 240 minutes [13-15,48].

Intraoperative Blood loss and Blood Transfusion

Intraoperative blood loss is an important risk factor in predicting AL [20]. Loss of blood during surgery may predispose to hypovolemia, resulting in tissue ischemia and impending anastomotic healing, which increases the risk of leakage [59]. In a meta-analysis conducted by Qu et al. involving 4580 rectal cancer patients, it was demonstrated that intraoperative blood loss >100 ml increased the risk of AL with OR 3.79 (95% CI: 2.48-5.49; P < 0.001) [41]. Leichtle et al. conducted a study including all cases of colectomy with primary anastomosis, which reported that intraoperative blood loss of more than 100 mL (OR 1.62; 95% CI: 1.10 to 2.40; p = 0.02) and 300 mL (OR 2.22; 95% CI: 1.32 to 3.76; p = 0.003) were associated with risk of developing AL [60]. Bleeding resulting in the need for blood transfusion is another risk factor [41]. Blood transfusion can lead to impairment of cell-mediated immune response, which increases the risk of infection around anastomoses and also impairs their healing [61]. In a retrospective study conducted by Park et al. including 1609 rectal cancer patients, it was shown that ≥2 units of perioperative blood transfusions were significantly associated with AL both on univariate (HR=6.030; p<0.001) and multivariate analysis (HR = 8.432; 95% CI: 4.715-15.185; P < 0.001) [20]. In another prospective multicenter study including 17,867 patients, Jannasch et al. found a 1.5-fold increased risk of anastomotic leak in patients undergoing blood transfusions, regardless of the number of blood units [17].

Diverting Stoma

After an anastomosis is constructed, diverting stoma is created to redirect the fecal flow away from the anastomotic site to help in anastomotic healing and protect from anastomotic failure [62]. However, there are controversial views regarding the link between a diversion stoma and anastomotic leakage. Matthiessen et al. conducted a randomized multicenter trial including 234 rectal cancer patients, in which it was demonstrated that the construction of a protective stoma had a significant association with decreased rates of AL. The leakage rate in patients with diverting stoma was 10.3% compared to 28.0% in patients without a stoma (OR = 3.4; 95% CI: 1.6-6.9; P < 0.001) [63]. In another meta-analysis study conducted by Huser et al., it was found that after surgery for low rectal cancers, there was a significant difference in the overall leakage and reoperation rates among patients with and without protective stomas with OR = 0.32 (95% CI 0.17-0.59) and OR = 0.27 (95% CI 0.14-0.51) respectively [64]. It is most beneficial when used selectively in high-risk patients with low pelvic anastomoses that are at an increased risk for AL. Contrary to the above studies, in a study done by Gastinger et al. including patients undergoing low anterior resection for rectal cancer, there was no difference in AL rates in patients with or without a stoma (14·5% vs. 14·2%, respectively; p=0.806) [65]. Similarly, Akiyoshi et al. conducted a study in Japan, including 363 rectal cancer patients who reported no significant association in AL rates in patients with (4.8%) and without stoma (3.3%) with a p-value of 0.4718 [66]. While this topic remains controversial, it is generally agreed that diverting stoma reduces the adverse effects of AL, such as fecal peritonitis, septic complications, and the need for repeat surgery if AL is to occur [67-69].

Prophylactic Drains

In colorectal surgery, there has been a lot of controversy surrounding the use of prophylactic drains. It was expected that the purpose of prophylactic drains was to eliminate perianastomotic fluid collections, thereby preventing abscess formation and reducing contamination that may otherwise extend to the anastomosis and cause AL [70]. A pelvic drain may also reduce the severity of leaks if they do occur, resulting in a less severe clinical outcome [41]. Prophylactic drains were also viewed as a potential early warning tool for anastomotic dehiscence [71]. In a retrospective study conducted by Peeters et al., it was concluded that the lack of pelvic drain after total mesorectal resection of rectal cancer was associated with an increased AL rate with a relative risk of 2.53 (95% CI: 1.57 -4.09; p <0.001) [72]. Also, a meta-analysis conducted by Rondelli et al., including 2277 patients, found a decreased AL rate with the use of pelvic drainage (OR = 0·51; 95% CI: 0·36 to 0·73) in patients undergoing anterior rectal resection with extraperitoneal colorectal anastomosis [73]. However, in contrast to the above assumptions, many studies claimed that using prophylactic drains was not associated with reducing AL rates [16,24,74,75]. A randomized trial conducted by Denost et al. reported that the use of pelvic drainage did not reduce AL rates [75]. In a meta-analysis conducted by Karliczek et al., it was concluded that after elective colorectal surgery the use of drains after colorectal anastomoses did not significantly improve outcomes in terms of wound infections, anastomotic dehiscence, reintervention, or mortality [74]. Despite the controversial conclusions, most surgeons use a drain in the abdominal or pelvic cavity following colorectal anastomotic surgery according to their personal preferences and own experiences [70].

Postoperative factors

Intestinal Microbes

The human gastrointestinal tract is inhabited by around 10^14 ^bacteria [76]. Some studies on animal models have suggested that alteration in intestinal flora appears to affect bowel anastomosis [77,78]. Cohn et al. demonstrated that intraluminal administration of antibiotics at an anastomotic site was capable of protecting a devascularized segment of the colon in their animal experiment [77]. In another study conducted by Shogan et al., it was found that cues released by surgically injured tissue can induce phenotypic changes in intraluminal microbes, thus making them pathogenic. It has been suggested that these may act as causative factors in the development of AL by increasing collagenase levels and activating host metalloproteinases [79]. Radiation is another powerful modality exerting a significant effect on gastrointestinal microflora by altering composition and virulence. It has been suggested that differential colonization could be linked to susceptibility to RT-induced diarrhea [80]. There is, nonetheless, a lack of evidence regarding the influence of gut microbiota on postoperative anastomotic complications.

Postoperative diet

Early initiation of enteral nutrition is critical in postoperative recovery [57]. Early administration of oral feeding has several benefits: it increases regular bowel movements, which improves microcirculation and perfusion at the anastomosis site, enhances anastomotic healing, and also prevents intestinal bacterial overgrowth [81]. In a retrospective study conducted by Jasarovic et al., including 153 colorectal cancer patients, it was reported that patients who started their oral intake in the immediate postoperative period had reduced AL than those who began later [81]. Studies have shown that an early postoperative diet helps in the early recovery of bowel function and reduces the length of hospital stay [82,83] (Table 2).

**Table 2:** Risk factors of Anastomotic leakage (AL) - Overview of studies AL: anastomotic leakage; ASA score: American Society of Anesthesiologists score; LAR: low anterior resection; BMI: body mass index

Author	Year	Country	Type of Study	No. of anastomoses	Anastomotic Leakage %	Conclusion
Jasarovic et al. [75]	2020	Serbia	Retrospective study	153	9.80%	Early initiation of oral feeding during the postoperative period reduces the AL rates
Hamabe et al. [21]	2018	Japan	Retrospective study	296	8.10%	Men undergoing LAR were at high risk of AL
Jannasch et al. [17]	2015	Germany	Prospective study	17 867	11.90%	Smoking and alcohol use increase the risk of AL
Midura et al. [58]	2015	United States	Retrospective study	13 684	3.80%	A prolonged operation duration of > 3 hours increases the risk of AL
Qu et al. [41]	2015	China	Meta-analysis	4589	6.30%	Preoperative chemotherapy increases the risk of AL; Intraoperative blood loss > 100ml increases the risk of AL
Frasson et al. [24]	2015	Spain	Prospective study	3193	8.70%	BMI > 30 increases AL rates
Pommergaard et al. [19]	2014	Sweden	Meta-analysis	110 272	7.20%	The patient's age did not correlate with AL risk; Low rectal anastomosis increases AL rates
Bakker et al. [18]	2014	Netherlands	Retrospective Study	15 667	7.50%	Increasing patient age is associated with an increased risk of death after AL; Patients with ASA scores III and IV are at risk of AL
Chang et al. [43]	2014	Korea	Retrospective study	1437	6.30%	Preoperative chemoradiotherapy is not associated with an increased risk of AL
Kang et al. [31]	2013	United States	Retrospective study	72 055	13.68%	Preoperative weight loss and malnutrition increase AL
Park et al. [20]	2013	Korea	Retrospective study	1699	6.30%	Male sex is significantly associated with the risk of AL; Perioperative transfusion of ≥ 2 units of blood increases the risk of AL
Leichtle et al. [60]	2012	United States	Retrospective study	4340	3.10%	Intraoperative blood loss > 100ml is associated with an increased risk of AL
Slieker et al. [39]	2012	Netherlands	Prospective study	259	7.30%	Steroid use increases the risk of AL
Lin et al. [12]	2011	Taiwan	Retrospective study	999	5.30%	Age > 70 years the significant risk of AL
Akiyoshi et al. [66]	2011	Japan	Retrospective study	363	3.60%	No statistical difference in AL between patients with and without diverting stoma
Telem et al. [14]	2010	United States	Case-Control study	3501	2.60%	Preoperative albumin < 3.5 mg/dl increases the risk of AL
Choi et al. [48]	2010	Korea	Retrospective study	156	10.30%	The level of anastomosis < 5cm from the anal verge is associated with an increased risk of AL
Zhu et al. [32]	2010	China	Retrospective study	132	9.10%	Preoperative anemia and hypoproteinemia increase the risk of AL
Jung et al. [11]	2008	Korea	Retrospective study	1391	2.50%	Age > 60 years the significant risk of AL
Jestin et al. [49]	2008	Sweden	Case-Control study	1381	9.70%	The level of anastomosis ≤ 6cm from the anal verge increases the risk of AL
Buchs et al. [15]	2007	Switzerland	Prospective study	811	3.80%	The risk of AL increases 2.5 times for every unit increase in ASA score
Matthiessen et al. [63]	2007	Sweden	Randomized control trial	234	19.20%	Diverting stoma is associated with reduced AL rates
Choi et al. [37]	2006	China	Retrospective study	1417	1.80%	ASA scores III and IV increase AL
Lipska et al. [16]	2006	New Zealand	Retrospective study	541	6.50%	Men are at increased risk of AL.
Konishi et al. [13]	2006	Japan	Prospective study	391	2.80%	Long-term steroid use is associated with an increased risk of AL; Operative time > 4 hours increases AL rates
Peeters et al. [72]	2005	Netherlands	Retrospective study	924	11.6%	Pelvic drain is associated with lower AL rates
Biondo et al. [27]	2005	Spain	Retrospective study	208	5.70%	Patients with BMI > 30 were at high risk of AL
Sørensen et al. [33]	1999	Denmark	Retrospective study	333	15.90%	Smoking and alcohol use increase the risk of AL

Limitations

This study has some limitations. Our review article used only PubMed and Google Scholar databases. While the study included all procedures under colorectal surgery, most of the data were related to colorectal cancer.

## Conclusions

Anastomotic leak in colorectal surgery is a multifactorial complication associated with an increased morbidity and mortality rate. It has remained the most feared complication over the past several years, despite numerous studies and technological advances like robotic surgery, staplers, and other anastomotic techniques. The clinical significance of reviewing and summarizing the risk factors of AL is to identify high-risk patients. Awareness and understanding of these factors will provide an opportunity to offer more comprehensive preoperative patient counseling. They will also facilitate early detection of leaks allowing appropriate treatment to be tailored accordingly, resulting in better patient outcomes. Among preoperative factors, gender played a more important role than a patient's age. Obesity, smoking, and alcohol abuse were all found to be significantly associated with the risk of AL. Among obese patients, visceral fat was a more significant indicator than BMI. The risk of AL increased with each unit increase in ASA score. Preoperative albumin level was the most critical factor in evaluating the preoperative nutritional status, influencing AL. Patients with chronic steroid use were at a higher risk than those on short-term steroids. The effect of neoadjuvant chemoradiation on AL had conflicting results in the literature review. Mechanical bowel preparation did not show any significant impact on postoperative complications. Intraoperative factors play a crucial role in predicting AL. The level of anastomosis was the most important of all factors. Anastomosis ≤ 5cm from the anal verge was at higher risk of leakage. Assessment of intestinal perfusion using intraoperative fluorescence imaging was beneficial in reducing the rate of AL. Operative time >3 hours increased the risk of AL. Intraoperative blood loss requiring blood transfusion also played a significant role in AL. Even though the use of diverting stoma was controversial, it was beneficial in the anastomotic healing of low pelvic anastomosis. Prophylactic drains remain controversial despite their widespread use among surgeons. The intestinal microbes appear to play a role in AL, but there was a lack of solid evidence in the literature reviewed. Early initiation of enteral feeding has shown to be beneficial in anastomotic healing and early postoperative recovery. It is beneficial to conduct future studies to focus on controversial risk factors that have not been adequately explored. The effect of neoadjuvant chemoradiation on AL needs to be further analyzed and interpreted in the context of overall survival. The role of intestinal microbes and the impact of intraluminal administration of antibiotics need further investigation.
